# A preliminary study of roxadustat in the treatment of aplastic anemia patients with inadequate erythroid responses

**DOI:** 10.1007/s00277-024-05799-5

**Published:** 2024-05-22

**Authors:** Yimeng Shi, Yufei Zhao, Weiru Liang, Baohang Zhang, Rui Kang, Wenrui Yang, Xin Zhao, Fengkui Zhang

**Affiliations:** 1 State Key Laboratory of Experimental Hematology, Haihe Laboratory of Cell Ecosystem, Institute of Hematology & Blood Diseases Hospital, National Clinical Research Center for Blood Diseases, Chinese Academy of Medical Sciences & Peking Union Medical College, Tianjin, 300020 China; 2 Tianjin Institutes of Health Science, Tianjin, 301600 China

**Keywords:** Aplastic anemia, Roxadustat, Hemoglobin response, Inadequate erythroid responses

## Abstract

Some aplastic anemia(AA) patients only have partial hematological responses after immunosuppressive therapy. Failure to achieve complete normalization of blood counts, particularly hemoglobin, will reduce their quality of life. This open-label pilot study was conducted to evaluate the efficacy and safety of roxadustat in this setting. A total of 14 patients with AA who had inadequate erythroid response after immunosuppressive therapy were included in the study. The primary efficacy endpoint was hemoglobin response at week 8 after roxadustat treatment. The median duration of roxadustat therapy was 14 (4–30) weeks, with 12 patients receiving roxadustat for ≥ 8 weeks. At week 8, nine patients (9/14, 64.3%) had their hemoglobin rising for at least 15 g/L, with two patients (2/14, 14.3%) achieving normal hemoglobin levels. By the last follow-up, hemoglobin responses were observed in 10 patients (10/14, 71.4%), with 4 patients(4/14, 28.6%) having normal hemoglobin levels. Roxadustat was tapered or discontinued in four responded patients; one relapsed after 12 weeks of tapering, and three maintained their response. Four patients (4/14, 28.6%) experienced mild adverse effects during therapy. Roxadustat is safe and well tolerated by patients with AA. Treatment with the hypoxia-inducible factor prolyl hydroxylase inhibitor improves hemoglobin levels in AA patients with inadequate erythroid responses.

**Supplementary Information:**

The online version contains supplementary material available at 10.1007/s00277-024-05799-5.

## Introduction

Aplastic anemia (AA) is characterized by a paucity of hematopoietic stem and progenitor cells (HSPCs) and pancytopenia caused by the autoimmune attack on the bone marrow [1]. HSPCs might be destroyed, inhibited, or induced to undergo symmetric rather than asymmetric division, and deranged T cell immunity, and the abnormal production of some cytokines play an important role in the pathogenesis [2, 3]. Approximately 70% of patients respond to immunosuppressive therapy; however, some patients only have a partial hematological response and fail to achieve normalization of blood counts, including hemoglobin levels.

Inflammatory cytokines such as interferon-γ (IFN-γ), tumor necrosis factor-α (TNF-α), and IL-6 are markedly elevated in the bone marrow and peripheral blood of AA patients [3]. Despite having a good hematological response to immunosuppressive therapy, AA patients still have significantly more CD3 + IFN + and CD3 + TNF + lymphocytes than normal subjects [4]. Immunosuppression cannot eliminate excess cytokines from the bone marrow of responded patients, suggesting that persistent excess inflammatory cytokines may be involved in AA residual hematopoietic erythropoiesis, impairing erythroid recovery after immunosuppressive therapy.

Hypoxia-inducible factor prolyl hydroxylase inhibitor (HIF-PHI) prevents hydroxylation of HIF-α, allowing for the transcription and expression of erythropoiesis-related genes, such as those related to erythropoietin (EPO) production and iron transport [5–9]. In addition, HIF-PHI indirectly suppresses hepcidin synthesis in hepatocytes via the EPO-erythroferrone pathway, which mobilizes iron release from iron storage cells, such as macrophages and hepatocytes [8]. Roxadustat, an orally administered HIF-PHI, inhibits prolyl hydroxylase by mimicking 2-oxoglutarate, one of its substrates. This drug stabilizes HIF-α levels in a dose-dependent manner and increases endogenous EPO to nearly physiological levels in patients with chronic kidney disease (CKD) [10]. Previous studies have demonstrated that roxadustat effectively increases hemoglobin levels regardless of inflammation and reduces intravenous (IV) iron requirement during CKD treatment [11]. Through stabilization of HIF-α, roxadustat also corrects inflammatory anemia via increased EPO production and improved iron homeostasis [12]. In an animal model of cisplatin-induced acute kidney injury, roxadustat significantly normalized TNF-α, IL-6, and other inflammatory cytokines in circulation and kidney tissues [13]. Zhao et al. found that roxadustat significantly decreased serum levels of inflammatory cytokines such as IFN-γ, TNF-α, IL-6 in CKD patients [14]. These findings indicate that roxadustat may have a potential anti-inflammatory effect.

Roxadustat is currently approved for treating anemia in CKD in China, Japan, Chile, South Korea, Europe, and the UK [9, 15–18], and has shown efficacy in improving anemia in patients with chemotherapy and low-risk myelodysplastic syndrome (MDS) [9, 19]. Hence, we hypothesized that roxadustat might have a therapeutic effect in AA patients with inadequate erythroid responses to immunosuppressive therapy. In this study, we retrospectively analyzed the data of 14 patients treated with roxadustat and report the efficacy of roxadustat in improving hemoglobin levels in these patients.

## Methods

### Patients

This study included AA patients treated with roxadustat at Blood Diseases Hospital, Chinese Academy of Medical Sciences & Peking Union Medical College from December 2021 to March 2023. Eligible patients had a diagnosis of AA, partial hematological responses after immunosuppressive treatment, inadequate erythroid responses after at least 6 months of standard-dose cyclosporine (CsA) ± androgen treatment, with stable peripheral blood parameters for at least 3 months. Inadequate erythroid response was defined as hemoglobin ≤ 100 g/L in AA patients who achieved partial hematological responses. In this study, AA diagnosis, severity, and immunosuppressive treatment efficacy were defined according to the literature [20–22], and previous immunosuppressive treatment for severe AA (SAA) was anti-human T lymphocyte porcine immunoglobulin (p-ALG) and CsA, and for non-severe AA (NSAA) patients CsA ± androgen.

Exclusion criteria included a history of an RBC transfusion or erythropoietin-stimulating agents within 4 weeks of enrollment; alanine aminotransferase (ALT) > 1.5×upper limit of normal (ULN), or aspartate aminotransferase (AST) > 1.5×ULN, or total bilirubin (TBIL) > 1.5×ULN, serum creatinine > ULN; anemia due to other etiologies such as iron deficiency, vitamin B12 or folate deficiency, hemolytic paroxysmal nocturnal hemoglobin (PNH); clinically significant or uncontrolled ongoing inflammatory/autoimmune/infectious disease; human immunodeficiency virus (HIV), hepatitis B virus (HBV), or hepatitis C virus (HCV) positivity; and clinically significant heart, liver, or kidney disease.

This study was approved by the Ethics Committee of the Institute of Hematology and Blood Diseases Hospital, Chinese Academy of Medical Sciences and Peking Union Medical College. All procedures were performed in accordance with the Helsinki Declaration of 1975, as revised in 2008. Written informed consent was obtained from all the patients and/or their legal guardians.

### Roxadustat treatment and efficacy criteria

Based on the weight of patients, the initial oral dose of roxadustat was 50 mg every other day for those <60 kg or 50 mg per day for those ≥ 60 kg. Roxadustat was tapered to discontinuation if the hemoglobin levels normalized or remained elevated and persisted for at least four weeks. All other therapies remained unchanged during roxadustat treatment.

The primary efficacy endpoint was hemoglobin response at the end of week 8 after roxadustat treatment. A hemoglobin response to roxadustat was defined as an increase in the hemoglobin level by 15 g/L from baseline, which was determined using the most recent peripheral blood count before roxadustat application. Hemoglobin ≥ 120 g/L in males and ≥ 110 g/L in females were defined as hemoglobin returned to normal. The last follow-up date was August 15, 2023.

### Statistical analysis

SPSS software (version 26.0) was used for the statistical analysis. Normally distributed measurements were expressed as mean ± standard deviation, and comparisons between groups were made using the t-test or ANOVA. Non-normally distributed measurements were expressed as median (range), and comparisons between groups were made using nonparametric tests. Count data were expressed as cases (percentage). All tests were two-tailed, and a *p-value* < 0.05 was considered statistically significant. The Kaplan–Meier product limit method was used to estimate the median time to hemoglobin response, and 95% confidence intervals (CIs) were assessed using the Brookmeyer and Crowley methods. Graphs were drawn using the GraphPad Prism 8 (La Jolla, CA).

## Results

### Characteristics of patients

A total of 14 AA patients with inadequate erythroid responses after immunosuppressive therapy were included in the study, eleven males and three females, including 13 NSAA and 1 SAA, with a median age of 32 (7–69) years, a median roxadustat treatment duration of 14 (4–30) weeks and a median follow-up of 18 (4–52) weeks. No patient required transfusion before roxadustat treatment. Of the 13 NSAA patients, 10 had been treated with CsA + androgen for 35 (17–186)months, and 3 were treated with CsA for 26 (16–111) months. The SAA patient was initially treated with p-ALG + CsA for 5 days and then received CsA + androgen for 11 months. The median hemoglobin before roxadustat treatment was 88 (62–99)g/L. The basic characteristics of patients before roxadustat treatment are presented in Table 1. The details of patient characteristics at diagnosis are provided in Table S1 in the Supplementary Appendix.

**Table 1:** Baseline characteristics of the patients before roxadustat treatment

Characteristics	Patients(*n* = 14)
Age(years), median(range)	32(7–69)
Male sex, no.(%)	11(78.6%)
Time since diagnosis(years), median(range)	3.6(0.9–32.8)
Duration of immunosuppressive therapy(months), median(range)	25(11–186)
Time since hematologic responses(months), median(range)	16(7–90)
Laboratory values, median(range)	
White blood cell count (×10^9^/L)	3.24(2.34–6.44)
Hemoglobin (g/L)	88(62–99)
Platelets (×10^9^/L)	43(30–148)
Reticulocyte count (×10^9^/L)	62.7(33.5-119.2)
ALT (U/L)	24.2 (15.6–39.7)
AST (U/L)	14.5 (10.9–30.0)
serum creatinine (umol/L)	74.7 (34.8–99.8)
PNH clone, no. (%)	
≥1%	2(14.3%)
<1%	12(85.7%)

The paroxysmal nocturnal hemoglobinuria(PNH) clone was defined according to the percentage of glycosylphosphatidylinositol-deficient neutrophils, as assessed using standard flow cytometry. ALT: alanine aminotransferase; AST: aspartate aminotransferase

### Hemoglobin response

All 14 patients completed 4 weeks of roxadustat treatment, with 6 patients (6/14, 42.9%) achieving hemoglobin responses. The median hemoglobin 100 (60–117) g/L at the end of 4 weeks of roxadustat treatment was significantly higher than that at baseline of 88 (62–99) g/L (*P* = 0.005).

Twelve patients received roxadustat for at least 8 weeks, and ten received treatment for ≥ 12 weeks. At 8 weeks of treatment, another three patients achieved hemoglobin responses, and two reached their normal hemoglobin levels. At the end of week 8, the cumulative probability of a hemoglobin response was 64.3% (9/14), and the hemoglobin normalization rate was 14.3% (2/14). At 12 weeks of treatment, another one patient achieved a hemoglobin response, and another one had a normal hemoglobin level. At the end of week 12, the cumulative probability of a hemoglobin response was 71.4% (10/14), and the cumulative rate of hemoglobin normalization was 21.4% (3/14). The median hemoglobin levels were 106 (45–123) g/L and 116 (33–126) g/L after 8 and 12 weeks of roxadustat treatment, respectively. With the extension of treatment, the increase in hemoglobin levels slowed.

By the last follow-up, one more patient had achieved a normal hemoglobin level at the end of 32 weeks of roxadustat treatment. Ten patients (10/14,71.4%) had achieved hemoglobin responses, with four of them (4/14,28.6%) reaching the normal range. The median time to achieve hemoglobin responses was 7.6 weeks (95% CI:5.7 to 9.5).

Compared to baseline, there were no significant changes in white blood cell (WBC) and platelets (PLT) counts after 4, 8, and 12 weeks of treatment or at the last follow-up (*P* > 0.05).

Of the four patients who did not achieve hemoglobin responses, one developed hemolytic PNH at four weeks of treatment and discontinued roxadustat treatment. The hemoglobin level in another patient fluctuated at 90 g/L without significant improvement during the 12 weeks of roxadustat treatment. A third patient had a progressive decrease from 62 g/L to 33 g/L in hemoglobin levels, which was considered a relapse of AA based on blood count changes and bone marrow examination. The fourth patient discontinued the drug autonomously at 8 weeks, and the hemoglobin level remained stable after drug discontinuation, with a hemoglobin level of 85 g/L at 44 weeks of follow-up.

**Fig. 1:**
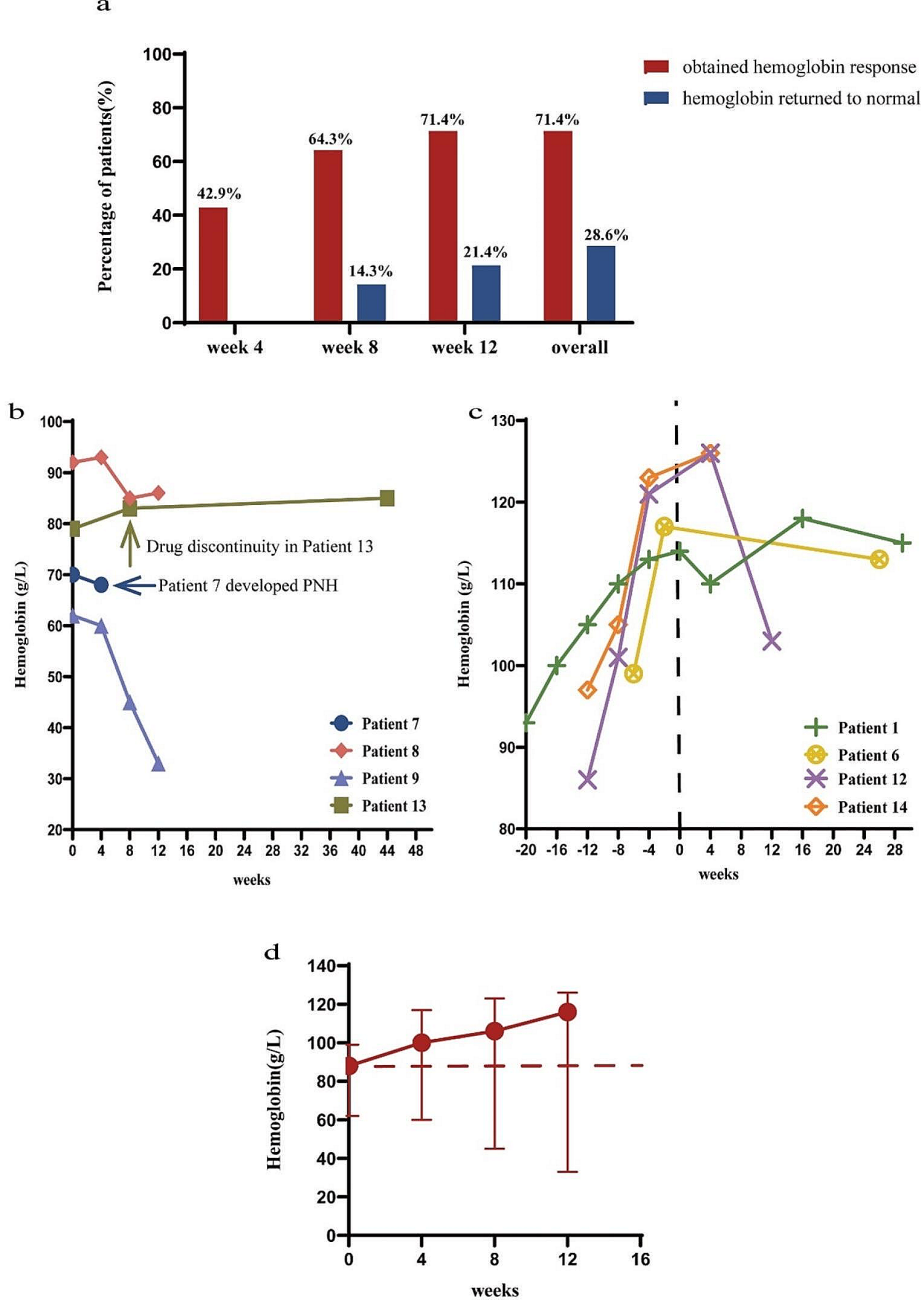
**Hemoglobin responses during roxadustat therapy.** The bar graph showed the hemoglobin responses and hemoglobin normalization rates at 4,8, and 12 weeks, respectively(**a**). Changes in hemoglobin levels during therapy in 4 non-responders(**b**). Changes in hemoglobin levels before and after drug discontinuation or tapering in 4 responders. Week 0 denotes when roxadustat was discontinued or tapered(**c**). Mean hemoglobin levels over time to week 12(**d**)

Hemoglobin responses during roxadustat therapy is shown in Fig. 1.

### Factors associated with efficacy

In this study, 12 patients completed 8 weeks of roxadustat treatment, and we compared the characteristics between responders and non-responders. Factors that may have affected the responses to roxadustat at week 8 are presented in Table 2. In hemoglobin responded and non-responded patients, the median disease duration was 2.1 (0.9–15.8) and 11.8 (3.3–32.8) years, median WBC 3.24 (2.65–6.44)×10^9^/L and 2.91 (2.34–4.40)×10^9^/L, median hemoglobin 88 (71–97)g/L and 86 (62–93)g/L, median PLT 51 (34–148)×10^9^/L and 38 (30–65)×10^9^/L, median reticulocyte counts 62.7 (42.0–107.3)×10^9^/L and 49.7 (33.5–64.7)×10^9^/L, median ALT 24.1 (16.0-39.7) U/L and 21.5 (15.6–24.7) U/L, median AST 14.1 (9.5–30.0) U/L and 15.1 (12.0-21.2) U/L, and median serum creatinine 70.4 (37.7–99.8) umol/L and 76.7 (68.8–90.0) umol/L, respectively. No factors associated with efficacy were found in the study (*P* > 0.05).

**Table 2:** Factors that possibly affected the responses to roxadustat

Characteristics	Responders(*n* = 8)	Non-responders(*n* = 4)
Age(years), median(range)	32(7–69)	29(19–62)
Male sex, no.(%)	6(75%)	3(75%)
Time since diagnosis(years), median(range)	2.1(0.9–15.8)	11.8(3.3–32.8)
Laboratory values, median(range)		
White blood cell count (×10^9^/L)	3.24(2.65–6.44)	2.91(2.34–4.40)
Hemoglobin (g/L)	88(71–97)	86(62–93)
Platelets (×10^9^/L)	51(34–148)	38(30–65)
Reticulocyte count (×10^9^/L)	62.7(42.0-107.3)	49.7(33.5–64.7)
ALT (U/L)	24.1 (16.0-39.7)	21.5 (15.6–24.7)
AST (U/L)	14.1 (9.5–30.0)	15.1 (12.0-21.2)
serum creatinine (umol/L)	70.4 (37.7–99.8)	76.7 (68.8–90.0)
PNH clone ≥ 1%, no.(%)	1(12.5%)	0(0%)

ALT: alanine aminotransferase; AST: aspartate aminotransferase; PNH: paroxysmal nocturnal hemoglobinuria

### Efficacy maintenance and relapse

Roxadustat was tapered to 50 mg every other day in three patients with stabilized normal hemoglobin for at least 1 month. Hemoglobin levels in the two patients were maintained within the normal range at the most recent follow-up. However, in the other patient, the hemoglobin level stabilized in the normal range after 4 weeks of roxadustat tapering but decreased by approximately 20 g/L after 12 weeks of tapering, except for the occurrence of PNH and AA relapse. The patient who spontaneously discontinued treatment at week 6 showed no significant change in hemoglobin 26 weeks after drug discontinuation. The median time to maintain of efficacy was 15 (4–29) weeks in the four patients.

### Adverse events and toxic effects

Overall, four patients (28.9%) experienced adverse events; one patient (7.1%) had a mild elevation of aminotransferase, two patients (14.3%) had a mild elevation of creatinine, and one patient (7.1%) had mild abdominal pain. All adverse events were considered mild, and the roxadustat dosage was not adjusted. One patient (7.1%) discontinued the drug after 4 weeks of treatment because of an increase in glycosylphosphatidylinositol (GPI)-deficient neutrophils from 17.3 to 58.8% and significant evidence of hemolysis. No deaths or progression to MDS or acute myeloid leukemia were detected during treatment with roxadustat.

## Discussion

In this study, we used roxadustat to treat AA patients with inadequate erythroid responses after immunosuppressive therapy. The results showed that the roxadustat administration promoted recovery of erythropoiesis and rapidly improved hemoglobin levels in this group of patients. The median time to achieve hemoglobin responses was 7.6 weeks, with more than 60% of the patients responding at 8 weeks of treatment. More patients responded with the extension of roxadustat therapy, and about 20% increased to their normal hemoglobin levels. We also noticed that the efficacy of roxadustat in the treatment of AA patients with inadequate erythroid response could be maintained for a relatively long time, even after drug withdrawal or tapering.

In addition to increasing endogenous EPO levels, roxadustat indirectly downregulated serum hepcidin levels, increasing iron availability [12, 23]. Roxadustat also ameliorates inflammatory anemia and exerts anti-inflammatory effects owing to its effects on HIF [10]. We did not conduct a detailed study on the mechanism of roxadustat in the treatment of AA patients; however, it is less likely that this drug exerts a therapeutic effect on correcting anemia in AA patients through increased EPO levels, as more than 60% of AA patients achieved hemoglobin responses with normal renal function. Therefore, roxadustat is more likely to improve hemoglobin levels in AA patients by increasing iron availability and inhibiting residual inflammatory cytokines. Further studies are required to understand the mechanism of action of roxadustat in treating AA patients with inadequate erythroid responses.

Roxadustat showed great therapeutic potential for correcting anemia; however, this study found no significant effect on the WBC and PLT counts. The therapeutic efficacy of this HIF-PHI may be limited to improving hemoglobin levels in AA patients, with little influence on the recovery of granulocyte and megakaryocyte lineages.

Compared with those who responded, non-responders appeared to have a longer duration of AA. For AA patients with a long history of the disease and stable peripheral blood parameters, the inadequate erythroid responses may be attributable to quantitative and functional abnormalities in HSPCs. Residual inflammatory cytokines may have a limited influence on hematopoietic recovery; therefore, roxadustat may be less effective in these AA patients. Due to the small sample size, we did not identify factors associated with efficacy, which needs further investigation in larger sample studies.

In the present study, the efficacy of roxadustat was maintained for a relatively long period after drug tapering for up to 29 weeks. After 12 weeks of drug tapering, the hemoglobin levels in one patient dropped significantly, which may be attributed to insufficient immunosuppression. Reactivation of abnormal immunity may decrease peripheral blood parameters, suggesting that sufficient immunosuppressive therapy remains the key in the treatment of AA.

Previous studies have demonstrated that adverse reactions of roxadustat in patients with CKD include diarrhea, vomiting, peripheral edema, headache, back pain, fatigue, and hyperkalemia [10]. In our study, adverse events were reported in 28.6% of the patients, including gastrointestinal discomfort and elevated aminotransferase and creatinine levels, which were mild and tolerable. Roxadustat is safe and well tolerated in AA patients with inadequate erythroid responses. Notably, roxadustat has off-target effects on CP4H substrates. Roxadustat suppresses the secretion of complement C1q; therefore, long-term use may reduce C1q levels [24]. Another study showed that this HIF-PHI significantly inhibited the hydroxylation and secretion of high-mol-weight forms of Mannose-binding lectin (MBL) [25]. MBL activates the lectin pathway of the complement system by co-opting MBL-associated serine proteases, thus playing a critical role in innate immune protection against several pathogens [26]. Some studies have revealed that MBL deficiency is associated with exacerbations and increased colonization of some pathogens, such as hepatitis B virus [27]. Whether roxadustat increases the incidence of infections and the long-term safety in AA patients should be assessed in future studies with larger sample sizes and longer follow-up durations.

A thrombopoietin receptor agonist (TPO-RA) may potentially benefit the treatment of anemia in AA patients with inadequate erythroid responses. However, because TPO-RA is not currently approved for the treatment of NSAA in China, options for managing anemia in AA patients, especially NSAA, are limited. Our findings confirmed the efficacy of roxadustat in treating anemia in AA patients with inadequate erythroid responses and provided a theoretical foundation for the use of this drug in AA.

This study showed that the application of roxadustat significantly increased hemoglobin levels in AA patients with inadequate erythroid responses after immunosuppressive therapy, and the efficacy was durable for up to 29 weeks. Overall, roxadustat was safe and well-tolerated by AA patients in this study. Given the small sample size included in this study and the relatively short follow-up period, our results might have been biased. Further prospective studies are need to provide more information on the efficacy and safety of roxadustat.

### Electronic supplementary material

Below is the link to the electronic supplementary material.

Supplementary Material 1

## Data Availability

No datasets were generated or analysed during the current study.
