# Supplementary material for: A preliminary study of roxadustat in the treatment of aplastic anemia patients with inadequate erythroid responses
Source: Ann Hematol. 2024 May 22;103(8):2757–63. doi: 10.1007/s00277-024-05799-5 (PMC11283381; doi:10.1007/s00277-024-05799-5)
Supplement: Supplementary file 1 — Supplementary Material 1 [file 277_2024_5799_MOESM1_ESM.docx]

**Supplementary Appendix**

Supplementary appendix for “A Preliminary Study of Roxadustat in the Treatment of Aplastic Anemia Patients with Inadequate Erythroid Responses” by Yimeng Shi^1,2^, Yufei Zhao^1,2^, Weiru Liang^1,2^, Baohang Zhang^1,2^, Rui Kang^1,2,^ Wenrui Yang^1,2^, Xin Zhao^1,2^, Fengkui Zhang^1,2*^

1 State Key Laboratory of Experimental Hematology, National Clinical Research Center for Blood Diseases, Haihe Laboratory of Cell Ecosystem, Institute of Hematology ＆ Blood Diseases Hospital, Chinese Academy of Medical Sciences ＆ Peking Union Medical College, Tianjin 300020, China

2 Tianjin Institutes of Health Science, Tianjin 301600, China

*Corresponding author

Table S1 Details of patient characteristics at diagnosis

| Patient ID | Sex | Age(years) | Diagnosis | Time since diagnosis(years) | Laboratory values at diagnosis | | | | | | | | | PNH clone | Transfusion requirement |
| --- | --- | --- | --- | --- | --- | --- | --- | --- | --- | --- | --- | --- | --- | --- | --- |
|  |  |  |  |  | WBC (×10^9^/L) | HGB (g/L) | PLT (×10^9^/L) | ARC (×10^9^/L) | ALT (U/L) | AST (U/L) | serum creatinine (umol/L) | Ferritin(ng/ml) | EPO(mIU/ml) |  |  |
| 1 | F | 38 | NSAA | 3.3 | 2.89 | 100 | 33 | 62.2 | 19.60 | 20.70 | 77.40 | 100.1 |  | 0% | NO |
| 2 | M | 45 | NSAA | 2.3 | 2.84 | 97 | 27 | 52.7 | 21.10 | 23.00 | 62.40 |  |  | 0% | NO |
| 3 | M | 25 | NSAA | 2.0 | 3.56 | 93 | 82 | 62.4 | 24.40 | 13.10 | 49.80 | 581.5 |  | 0% | NO |
| 4 | M | 32 | NSAA | 15.8 | 3.74 | 96 | 23 | 83.5 | 18.10 | 19.00 | 87.00 |  |  | 0% | NO |
| 5 | F | 36 | NSAA | 1.4 | 4.54 | 71 | 34 | 53.4 | 14.50 | 16.00 | 51.30 | 53.2 | ＞765 | 0% | NO |
| 6 | M | 18 | NSAA | 9.3 | 3.33 | 37 | 2 | 66.3 | 37.40 | 31.50 | 51.10 |  |  | 0% | YES |
| 7 | M | 45 | NSAA | 2.0 | 2.71 | 59 | 10 | 52.6 | 33.50 | 29.40 | 75.50 | 416.4 |  | 27% | YES |
| 8 | M | 62 | NSAA | 32.8 | 2.30 | 63 | 24 | 64.7 | 23.90 | 21.20 | 90.00 |  |  | 0% | YES |
| 9 | M | 20 | NSAA | 14.3 | 4.99 | 57 | 23 | 30.4 | 15.60 | 12.10 | 68.80 | 1500 |  | 0% | YES |
| 10 | M | 16 | NSAA | 7.5 | 2.99 | 67 | 33 | 74.5 | 16.10 | 20.50 | 35.90 |  |  | 0% | NO |
| 11 | M | 31 | NSAA | 3.8 | 3.62 | 85 | 34 | 64.5 | 21.20 | 18.10 | 85.50 |  |  | 1.3% | NO |
| 12 | M | 69 | NSAA | 2.0 | 3.46 | 103 | 28 | 87.7 | 24.90 | 26.50 | 101.80 |  |  | 0% | NO |
| 13 | M | 19 | NSAA | 9.3 | 2.61 | 72 | 31 | 58.7 | 42.60 | 26.30 | 51.40 |  | 758 | 0% | NO |
| 14 | F | 7 | SAA | 0.9 | 2.5 | 83 | 14 | 26.3 | 10.20 | 9.50 | 35.10 | 63 | 22.12 | 0% | YES |

The paroxysmal nocturnal hemoglobinuria(PNH) clone was defined according to the percentage of glycosylphosphatidylinositol-deficient neutrophils, as assessed using standard flow cytometry. WBC: white blood cell; HGB: hemoglobin; PLT: platelets; ARC: reticulocyte counts; ALT: alanine aminotransferase; AST: aspartate aminotransferase; EPO: erythropoietin; PNH: paroxysmal nocturnal hemoglobinuria; NSAA: non-severe aplastic anemia; SAA: severe aplastic anemia.
